# Deep sequencing reveals increased DNA methylation in chronic rat epilepsy

**DOI:** 10.1007/s00401-013-1168-8

**Published:** 2013-09-05

**Authors:** Katja Kobow, Antony Kaspi, K. N. Harikrishnan, Katharina Kiese, Mark Ziemann, Ishant Khurana, Ina Fritzsche, Jan Hauke, Eric Hahnen, Roland Coras, Angelika Mühlebner, Assam El-Osta, Ingmar Blümcke

**Affiliations:** 1 Department of Neuropathology, University Hospital Erlangen, Schwabachanlage 6, 91054 Erlangen, Germany; 2 Epigenetics in Human Health and Disease, Baker IDI Heart and Diabetes Institute, The Alfred Medical Research and Education Precinct, Melbourne, VIC Australia; 3 Epigenomics Profiling Facility, Baker IDI Heart and Diabetes Institute, The Alfred Medical Research and Education Precinct, Melbourne, VIC Australia; 4 Department of Pathology, The University of Melbourne, Parkville, VIC Australia; 5 Faculty of Medicine, Monash University, Melbourne, VIC Australia; 6 Center of Familial Breast and Ovarian Cancer, University Hospital of Cologne, Cologne, Germany; 7 Center for Molecular Medicine Cologne (CMMC), University of Cologne, Cologne, Germany; 8 Department of Pediatrics and Adolescent Medicine, Medical University Vienna, Vienna, Austria

**Keywords:** Epilepsy, Hippocampus, Epigenetic, DNA methylation, Massive parallel sequencing, Ketogenic diet

## Abstract

Epilepsy is a frequent neurological disorder, although onset and progression of seizures remain difficult to predict in affected patients, irrespective of their epileptogenic condition. Previous studies in animal models as well as human epileptic brain tissue revealed a remarkably diverse pattern of gene expression implicating epigenetic changes to contribute to disease progression. Here we mapped for the first time global DNA methylation patterns in chronic epileptic rats and controls. Using methyl-CpG capture associated with massive parallel sequencing (Methyl-Seq) we report the genomic methylation signature of the chronic epileptic state. We observed a predominant increase, rather than loss of DNA methylation in chronic rat epilepsy. Aberrant methylation patterns were inversely correlated with gene expression changes using mRNA sequencing from same animals and tissue specimens. Administration of a ketogenic, high-fat, low-carbohydrate diet attenuated seizure progression and ameliorated DNA methylation mediated changes in gene expression. This is the first report of unsupervised clustering of an epigenetic mark being used in epilepsy research to separate epileptic from non-epileptic animals as well as from animals receiving anti-convulsive dietary treatment. We further discuss the potential impact of epigenetic changes as a pathogenic mechanism of epileptogenesis.

**Electronic supplementary material:**

The online version of this article (doi:10.1007/s00401-013-1168-8) contains supplementary material, which is available to authorized users.

## Introduction

Approximately 0.5–1 % of people suffers from epilepsy. One frequent epileptic syndrome is associated with drug-resistant temporal lobe seizures and hippocampal neurodegeneration, i.e., temporal lobe epilepsy with hippocampal sclerosis (TLE-HS) [9]. Onset with an initial precipitating injury including trauma, inflammation, or prolonged acute symptomatic seizures is most characteristic, followed by a clinically silent latency period before progression of spontaneous recurrent seizures [7, 58]. This pathogenic time course can be recapitulated in experimental animal models of TLE, although specific disease mechanisms remain poorly understood. Array-based profiling studies have detected aberrant gene expression patterns. Several genes implicated in epilepsy have been identified and are thought to participate in inflammation and stress, synaptic transmission and signal transduction, ion transport, cell metabolism as well as synaptic plasticity [4, 16, 22, 26, 45]. We recently hypothesized that a common regulatory trait for these abundant and long-lasting changes in gene expression relates to epigenetic changes such as genomic DNA methylation [34, 35] as well as specific chromatin changes, including histone tail modification. Recent experimental studies provide some evidence for aberrant epigenetic signatures induced by seizures. For example, alterations in histone tail modifications have been reported for histones H3 and H4 [28, 30, 69, 73] as well as phosphorylation of histone variant H2A.X [14]. Furthermore, the neuron restrictive silencing factor (Nrsf), which regulates neuronal gene expression and recruits DNA methyltransferases (Dnmts) as well as histone deacetylases (Hdacs), is involved in seizure development and progression [21, 44, 51]. Altered microRNA expression [31] as well as DNA methylation patterns are also observed in rodent epilepsy models [53]. Our group was the first to show that aberrant DNA methylation of the Reelin promoter associated with granule cell dispersion in human TLE [36]. Furthermore, increased DNMT expression was observed in temporal neocortex samples obtained from TLE patients [79]. The experimental results of these studies implicate aberrant DNA methylation in the late, chronic and drug-resistant stage of the disease.

Herein we used a massive parallel sequencing approach to examine persistent changes in genomic CpG methylation correlated with gene expression in a chronic rat TLE model. Since dietary intake and nutrition are implicated in modifying epigenetic patterns [18], we examined the ketogenic diet (KD) in our model of chronic rat epilepsy. The high-fat, low-carbohydrate ketogenic diet is well recognized as anti-epileptic therapy with moderate success in man and experimental animal models [33, 38, 50, 54]. In this study, we demonstrate that early administration of an anti-convulsive ketogenic diet is associated with gene regulating DNA methylation changes in rat TLE.

## Materials and methods

### Animal model: surgery, induction of status epilepticus and video-EEG recording

The experimental study was approved by the local animal care and use committee in accordance with the European Communities Council Directive (54-2532.1-23/09, Directive 2010/63/EU). Male Wistar rats weighting 300–350 g (Charles-River, Germany; *n* = 14) were kept in individual cages under controlled environmental conditions (12 h dark/light cycles, 20–23 °C and 50 % relative humidity) with drinking and feeding ad libitum. Animals were either fed a standard or ketogenic diet (KD, 4:1 ratio of fat over protein and carbohydrates, specifically designed for rodents and distributed by Altromin, Lage, Germany; data sheet access via http://www.altromin.de/de/upload/media/specs/sonder/30009.pdf) and weekly controlled for body weight (Supplement Fig. 1a).

Representative animals were assigned to continuous video-electroencephalography monitoring (vEEG; *n* = 5; DSI, St. Paul, MN, USA). Electrodes were implanted before seizure induction. Rats were deeply anesthetized with an intraperitoneal injection of a ketamine (57 mg/kg) and xylazine (9 mg/kg) mixture and placed in a stereotaxic apparatus (Bilaney Consultants, Düsseldorf, Germany). The skull was exposed and two holes were drilled (2 mm lateral to the sagittal suture and 5 mm anterior to the lambda suture, 1 mm diameter) to insert stainless steel screws (1 mm diameter, DIN84, Hummer und Riess, Nuremberg, Germany) passing the bone and touching but not penetrating the dura. Screws were connected with polyimide coated stainless steel lead wire, serving as EEG electrodes. Afterwards, both screws were glued together with lead wires and surrounding bone using dental acrylic. The transmitter (F40-EET, DSI, St. Paul, MN, USA) was placed into a subcutaneous pocket along the animal’s dorsal flank. All animals had time to recover for 1 week before further procedures. There were no differences in baseline EEG between animals before further treatment.

To induce status epilepticus (SE), animals were injected with a single high dose of the muscarinic receptor agonist pilocarpine (PILO; 340 mg/kg, i.p.; Sigma-Aldrich, Steinheim, Germany). Peripheral cholinergic effects were minimized by administration of methyl scopolamine (1 mg/kg, s.c.; 30 min before injection of pilocarpine; TCI Europe NV, Zwijndrecht, Belgium). Animals that experienced no SE within 45 min after first pilocarpine administration were treated for a second time with half the application dosis (175 mg/kg, i.p.). After 60 min SE duration, animals received administration of diazepam (8 mg/kg, i.m.; Sigma-Aldrich). One hour following diazepam treatment glucose depots (2 × 5 ml) were subcutaneously injected to help animals to recover. Age matched control rats (CTRL; *n* = 5) received methyl scopolamine and saline (0.9 % NaCl; Sigma-Aldrich) injections only.

Behavioral seizures were scaled from 1 to 5 according to Racine [61]. SE was defined as minimum three Racine “class 5” behavioral seizures within 10 min and was identified, where applicable, from EEG recordings by high-amplitude and -frequency discharges. All rats were continuously monitored (video or vEEG, 24 h/7 days) following pilocarpine injections. The observation period lasted 84 days. Available EEGs (PILO *n* = 3/4 and PILO + KD *n* = 2/5) were automatically screened for spontaneous seizure activity using the NeuroScore software (DSI). An EEG seizure was defined as a period of consistent, repetitive changes in amplitude and frequency of electrical activity that persisted for more than 10 s (Supplement Fig. 1c). Video images were used to confirm a clinical seizure where EEG abnormalities were detected. Behavioural seizures were further evaluated for the following parameters: latency to the appearance of first spontaneous seizure after SE in days; seizure severity according to Racine’s scale; seizure duration in seconds; seizure frequency defined as the total number of detected seizures per week and life time seizures.

Neuropathological evaluation of our rat TLE model was performed in video-monitored reference cohorts (CTRL, PILO and PILO + KD; *n* = 3 each) treated as described above, but terminated 4 weeks following SE or sham injection. A detailed description of method and results is provided in the legend to Supplement Fig. 2.

### Tissue preparation

For tissue preparation ether anesthetized animals were decapitated, the sculls opened and overlaying cortex removed for preparation of dorsal hippocampus. Five-mm-thick hippocampal slices were prepared along the septo-temporal axis and collected in icecold PBS, thereby, removing blood contamination. Hippocampi were snap frozen in liquid nitrogen and stored at −80 °C until further use. Each probe was individually processed and not pooled with other samples. For downstream applications hippocampus was homogenized in ice cold 1× PBS and divided into equal volumes for DNA and RNA extraction.

### DNA methylation profiling

Ten mg rat hippocampal tissue was used for genomic DNA extraction using the DNeasy Blood and Tissue Kit (Qiagen, Hilden, Germany) according to the manufacturer’s instructions. Massive parallel sequencing of enriched methylated DNA was performed as described previously [57]. Briefly, 500 ng of rat hippocampal DNA from each animal (*n* = 14) was fragmented to a median size of 200–300 bp and subjected to methylated DNA capture according to the MethylMiner protocol (Invitrogen, Darmstadt, Germany), exclusively enabling capture of methylated double stranded DNA. Fragmented and enriched DNA was eluted at high salt concentrations (2 M NaCl). Ten ng of enriched DNA was used in library preparation using the NEBNext DNA Library Prep Reagent Set for Illumina (New England Biolabs, Frankfurt/Main, Germany). Quality of sequencing libraries was assayed using the Shimadzu MultiNA capillary electrophoresis system (Shimadzu, Kyoto, Japan). Libraries were sequenced at a concentration of 10 pM on the Illumina Genome Analyzer IIx (Illumina, San Diego, CA, USA) with a 36 bp single read length. Image analysis and base-calling were performed with OLBv1.8 software. Sequenced tags were aligned to the rat genome RN4 using BWA (version 0.5.9) with default alignment parameters [43]. Profiles of DNA methylation were compared between all pairwise combinations of samples using the MACS peak calling software (version 1.4.0 rc2) with a fixed shift size of 75 bp and a significance cut-off of 10E-05 [78]. Genomic regions showing different methylation patterns between pairs of samples were merged using Bedtools [59]. Duplicate reads which aligned to the same location in a given sample were removed from further analysis. The numbers of read tags aligning to each region were summarized using a custom python script producing a matrix of counts (tags per region per sample). The regions were non-differentially filtered for regions, where the sum of tag counts was below the 50th centile. These count data were tested for differential tag abundance using Bayesian shrinkage of a negative binomial model implemented in edgeR [64], and normalized using trimmed mean normalization [65]. An adjusted *p* value was calculated using the Benjamini–Hochberg false discovery rate (FDR) [6]. Filtered gene lists meeting our significance criteria were submitted to pathway analysis using resources supplied by the database for annotation, visualization and integrated discovery (DAVID) [27].

### Gene expression profiling

Ten mg rat hippocampal tissue was used for total RNA extraction using the Trizol method, followed by DNAse digestion. RNA quality was verified on the Shimadzu MultiNA capillary electrophoresis system (Shimadzu). Following Dynabead Oligo(dT) enrichment (Invitrogen), mRNA was prepared into sequence ready libraries with the NEBNext mRNA Library Prep Reagent Set for Illumina (New England Biolabs). These samples were sequenced as above. Sequenced tags were aligned to the rat genome RN4 using BWA (version 0.5.9) using default alignment parameters [43]. The numbers of read tags aligning to each gene were extracted using a custom python script producing a matrix of counts with regions based on the Ensembl transcript annotation (version 66). Genes were non-differentially filtered for tag counts sums below the 30th centile. This count data was tested for differential tag abundance using Bayesian shrinkage of a negative binomial model implemented for in edgeR [64], and normalized using trimmed mean normalization [65]. An adjusted *p* value was calculated using the Benjamini–Hochberg false discovery rate (FDR) [6].

### Gene set enrichment analysis of expression

Rank scores for differential mRNA expression were calculated as −log10 (*p* value) multiplied by the sign of the edgeR fold change so that upregulated genes had positive scores. These rank scores were used to test for correlations between mRNA expression and DNA methylation or ChIP-Seq derived gene sets using the GSEA preranked method based on 1,000 gene set permutations [72]. Sets of differentially methylated genes were derived by taking the differentially methylated regions for each pairwise comparison between PILO, and PILO + KD with CTRL filtered for a *p* value <0.01 as determined by edgeR analysis, and separated into increased and decreased methylation. If any of these regions were co-located with either a gene body, TSS, promoter (−3 kb from TSS), exon or intron they were assigned to that gene set, e.g., genes with an exon overlapping a region of increased methylation. The clustered transcription factor data set was downloaded (ftp://hgdownload.cse.ucsc.edu/apache/htdocs/goldenpath/hg19/encodeDCC/apache/htdocs/goldenpath/hg19/encodeDCC/wgEncodeRegTfbsClustered/) from the ENCODE profiling project [17]. A gene set for each transcription factor and cell line was generated by intersecting ChIP-Seq data with promoter annotation (−3 kb from TSS) using Ensembl human genes (version 66), and assigning the intersecting gene promoters to that gene set. This analysis generated 425 gene sets, of which 403 (depending on gene set size) were used in GSEA. Rat genes were mapped to Human genes using Homologene [1].

### Quantification of gene expression

Total RNA was isolated using TRIzol (Invitrogen). Genomic DNA contamination was removed by DNAse treatment (Qiagen). First-strand cDNA synthesis was performed using the SuperScript II first strand synthesis Kit (Invitrogen) according to the manufacturer’s instructions. Gene expression was analyzed on an ABI Prism 7500 Fast Real-Time PCR Detection System (Applied Biosystems, Foster City, CA, USA). Forward and reverse primers were used at 100 nM together with Power SYBR Green Master Mix (Applied Biosystems). Reactions were incubated at 95 °C for 10 min, followed by 40 cycles of 95 °C for 10 s and 60 °C for 30 s. For relative quantification (comparative ΔΔCt method), gene expression was assessed at least in triplicates and normalized to internal reference Actb and Gapdh. cDNA specific primers were designed as follows: *Camkk2* fw-AGAACTGCACACTGGTCGAG, rev-CCGGCTACCTTCAAATGGGT; *Il10rb* fw-CTGGAGCCATGGACAACTTACT, rev-GGAGGGGTTGTTTCATCACTG; *Actb* fw-GAGAAGAGCTATGAGCTGCC, rev-TCCATACCCAGGAAGGAAGG; Gapdh fw-GGCTGGCATTGCTCTCAATG, rev-CATGTAGGCCATGAGGTCCA.

### Bisulfite sequencing

DNA samples were processed for methylation-specific sequencing as described previously [25, 36]. Briefly, 1 μg of genomic DNA was bisulfite converted using EpiTect DNA Bisulfite Kit (Qiagen), the region of interest pre-amplified, subcloned using topoisomerase TA vector (Invitrogen) and white colonies were selected and grown in LB broth. Plasmid was purified using Gene Jet Plasmid Miniprep Kit (Fermentas, St. Leon-Rot, Germany) and clones sequenced (commercial sequencing facility of GATC Biotech, Konstanz, Germany). A minimum of six clones per subject with proven insertion of the PCR fragment were analyzed. Sequences were quality controlled and aligned using the CLC sequence viewer v. 6.3 (CLC bio) and Quantitation tool for Methylation Analysis software (RIKEN). Primers used for bisulfite sequencing were the following: *Camkk2_*BIS fw-TTTAGAGGGGATTTGAGTTTTT, rev-ATCCACCAATAAATCCAAATATTAC; *Il10rb*_BIS fw-GGGTTAGGATTGAGTTTGTAGAT, rev-TAAATAATAAAACCACCAAATTTATACTC.

### Data access

Methyl-Seq and mRNA-Seq data were deposited in the NCBI Gene Expression Omnibus (GEO; http://www.ncbi.nlm.nih.gov/geo/query/acc.cgi?acc=GSE50080).

## Results

### Massive parallel sequencing identifies distinct genomic DNA methylation profiles in chronic rat epilepsy compared with controls

**Fig. 1:**
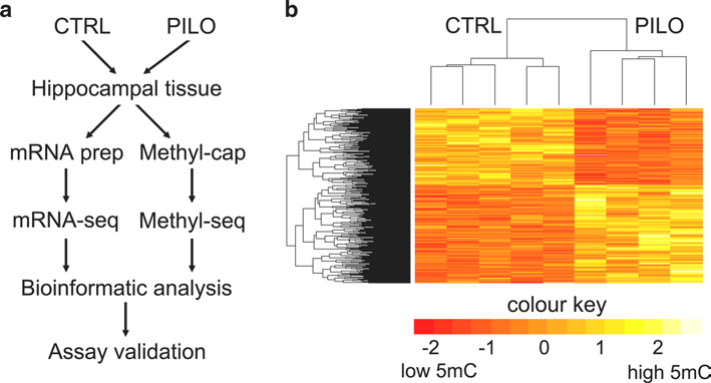
Deep sequencing (Methyl-Seq) revealed increased genomic DNA methylation in chronic rat epilepsy. **a** Study design, **b** heat map displaying hierarchical clustering of samples and genomic regions according to differential methylation profiles (*yellow* methylation up, *red* methylation down). A specific DNA methylation signature characterized chronic rat epilepsy. *CTRL* sham injected, healthy controls; *PILO* pilocarpine injected, chronic epileptic animals. Clustering was performed by taking the trimmed mean normalized values for differential regions as defined by edgeR analysis with a *p* value <0.01. These values were normalized to the standard normal distribution before performing Euclidean distance based hierarchical clustering on both regions and samples using the heatmap.2 function in the R package gplots

DNA methylation is a major epigenetic regulator of gene suppression [52], and has been implicated in experimental and human temporal lobe epilepsies [36, 53, 79]. However, our knowledge on genomic methylation mediating gene expression changes in epilepsy is limited (1) to few specific gene loci comprehensively analyzed and (2) to the early stage of the disease (during or immediately following SE). To examine the role of genomic DNA methylation in the chronic epileptic state, we used Methyl-capture and massive parallel sequencing (Methyl-Seq) of hippocampal tissue obtained from rats 12 weeks following pilocarpine induced status epilepticus (PILO, *n* = 4) and compared these with respective healthy controls (CTRL, *n* = 5). We also examined gene expression changes using mRNA sequencing (mRNA-Seq) from the same tissue specimens to analyze the biological relevance of methylation changes (Fig. 1a).

Whole-genome DNA methylation profiling was performed as previously described [12, 57]. A minimum of 15 million short single reads (36 bp) was sequenced per sample, and an average 83 % of the short reads mapped unambiguously to the rat genome (RN4). The other 17 % of reads were likely to originate from repetitive DNA. Inter-individual sample comparison revealed 310,070 regions covering 203,265,410 bp with variant methylation (~7.3 % of the rat genome). Pairwise comparison of global DNA methylation detected strong differences in methylation patterns between all CTRL and PILO samples. Hierarchical cluster analysis was used to assess and visualize underlying differences in DNA methylation profiles between samples and phenotypes. As shown in Fig. 1b, DNA methylation profiles readily discriminated samples into discrete groups according to the treatment.

**Fig. 2:**
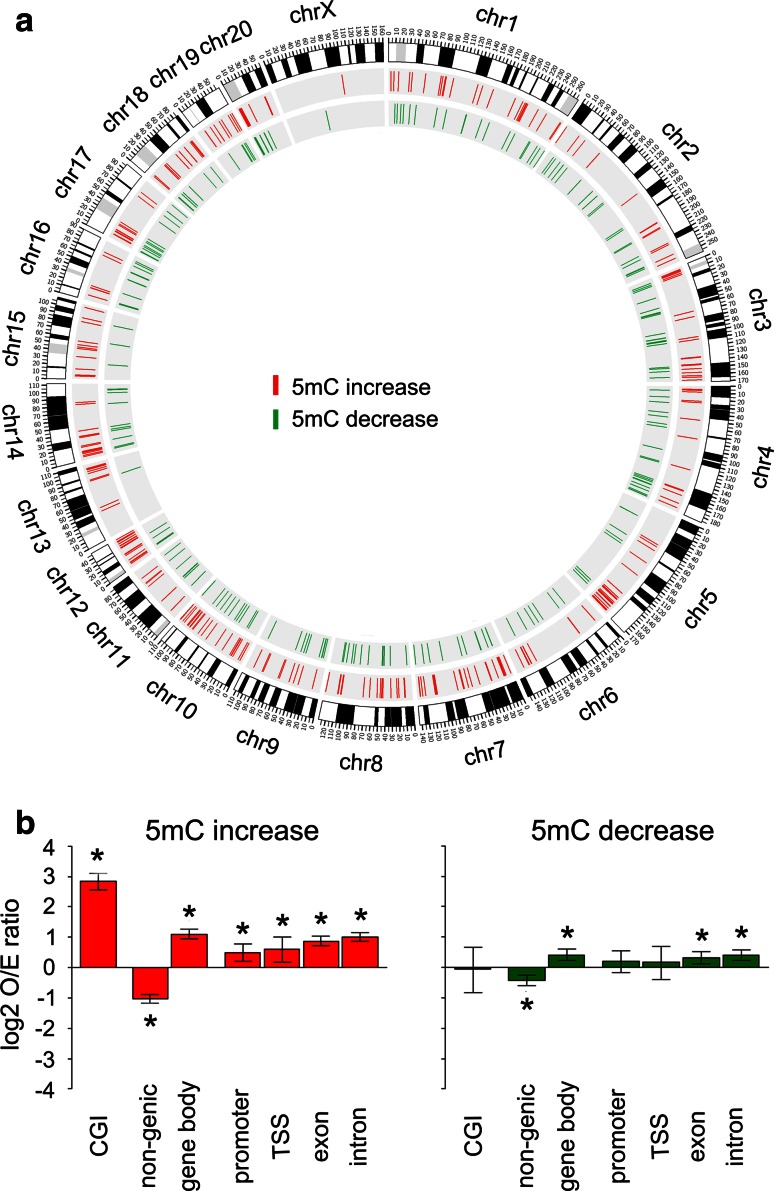
Genomic distribution of DNA methylation changes (cut-off *p* < 0.01) in chronic rat epilepsy. **a** Rat genome ideogram summarizing hypermethylation (*red*) and hypomethylation events (*green*) in PILO versus CTRL. DNA methylation targeted the entire genome with almost complete sparing of the X-chromosome (ChrX). **b** Frequency of observed methylation changes compared to non-differentially methylated regions [−log10 (*p* value) <0.25], with upper and lower 95 % confidence intervals for different genomic features. Hypermethylation relative to controls is shown in the *left panel* (*red bars*), whereas, hypomethylation relative to controls is shown in the *right panel* (*green bars*). DNA methylation events were mainly confined to CGIs, but did not frequently target promoters. *CGI* CpG island, *TSS* transcriptional start site, *O/E* observed/expected ratio, *5mC* 5-methyl-cytosin. *Asterisks* indicate significance (*p* < 0.05 using Fisher’s Exact test)

To further examine changes in DNA methylation and its association with phenotype we mapped methylation profiles to the rat genome using Circos [39]. Increased (hyper-) and decreased (hypo-) methylation in PILO samples were assigned to each chromosome and shown in red and green, respectively (Fig. 2a). We observed phenotype-specific regionalization of hyper- and hypomethylation events, which targeted the entire genome except chromosome X. Our analysis identified 2,573 individual loci that discriminate between chronic epileptic and control animals (cut-off *p* < 0.01). Thereof, 1,452 loci were hypermethylated and 1,121 loci hypomethylated in chronic epilepsy samples compared to controls. These data imply that DNA methylation patterns of PILO animals are indeed distinguishable from the reference CTRL animals.

### Differential DNA methylation in chronic rat epilepsy is mainly confined to gene bodies

Because hypermethylation of CpG-rich promoters is commonly regarded as a strong indicator of gene suppression in physiologic and pathologic conditions [77], we examined the extent of DNA methylation at different genomic regions. We compared the distribution of genomic methylation at CpG islands (CGI), non-genic and genic regions (gene body including 5′ and 3′ untranslated regions), promoters (3 kb upstream of transcription start), transcriptional start sites (TSS) and exons as well as introns in the dissected hippocampi of chronic epileptic animals relative to controls. Differentially methylated regions were normalized and expressed as log2 odds ratios of observed differences compared to non-differentially methylated genomic features. We report the mean frequency of methylation changes with upper and lower 95 % confidence intervals for the genomic features (Fig. 2b). We identified a significant increase in DNA methylation content in chronic epilepsy specimen, when comparing hyper- and hypomethylation events at specific genomic features. Table 1 shows predominant increase in DNA methylation at CGIs and gene bodies, exons as well as introns (Fisher’s Exact Test, *p* < 0.05). Generally, DNA methylation changes were frequently found at CpG islands (CGI) as well as genic and non-genic regions. Regarding non-genic sites, we did not so much observe differential methylation of gene promoters (Fig. 2b). Instead, we detected changes more readily distal from coding regions of genes.

**Table 1:** Genomic features targeted by DNA methylation

Feature	Up_OL	Up_NOL	Down_OL	Down_NOL	log2 odds ratio	Lower CI	Upper CI	*p* value
CGI	148	1,304	17	1,104	2.881	2.142	3.708	9.7E-22
Gene body	831	621	510	611	0.681	0.450	0.912	3.7E-09
Non-genic	692	760	651	470	−0.605	−0.836	−0.374	1.8E-07
Intron	783	669	489	632	0.597	0.366	0.828	2.3E-07
Exon	438	1,014	255	866	0.553	0.289	0.818	2.5E-05
Promoter	108	1,344	69	1,052	0.293	−0.173	0.766	2.1E-01
TSS	53	1,399	31	1,090	0.413	−0.264	1.113	2.2E-01

Table shows statistics comparing ratio of the number of increased versus decreased methylated regions (cut-off *p* < 0.01) across genomic features. *p* value calculated with Fisher’s exact test (*p* < 0.05 was considered significant)

*CGI* CpG island, *TSS* transcriptional start site, *OL* number of loci overlapping feature (overlap ≥1 bp), *NOL* number of loci not overlapping feature, *CI* confidence interval

### Gene expression profiling in chronic epileptic rats

**Fig. 3:**
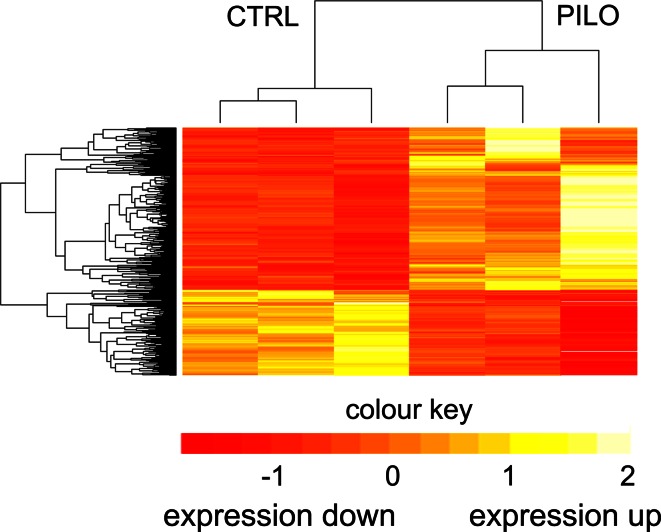
mRNA-Seq identified highly distinct gene expression signatures in chronic rat epilepsy and controls. Heat map displaying hierarchical clustering of samples and genes according to differential expression profiles normalized to the standard normal distribution (*yellow* expression up, *red* expression down). Treatment groups can be clearly differentiated by their expression profiles. *CTRL* sham injected, healthy controls; *PILO* chronic epileptic animals. Clustering was performed by taking the trimmed mean normalized values for genes as defined by edgeR analysis with a *p* value <0.01. These values were normalized to the standard normal distribution before performing Euclidean distance based hierarchical clustering on both regions and samples using the heatmap.2 function in the R package gplots

We have previously postulated DNA methylation changes as potential cause of aberrant gene expression in experimental and human TLE [34, 35]. To test this hypothesis, we compared genomic methylation patterns with gene expression data derived from same hippocampal specimens using mRNA-Seq (*n* = 3 per treatment group). Gene expression profiling identified 1,502 genes that were differentially expressed in chronic epileptic rats compared to healthy controls (cut-off *p* < 0.01; Fig. 3). Gene annotation in DAVID [27] revealed biological functions implicated in pathomechanisms underlying chronic epilepsy, which was consistent with previous reports from animal and human studies using epileptic brain tissue. KEGG pathway analysis of downregulated genes in chronic epileptic hippocampi (*n* = 517) showed enrichment for MAPK and calcium signaling, axon guidance, long-term depression and potentiation, whereas overexpressed genes (*n* = 985) were associated with pathways participating in chronic inflammation, immune response, and neurodegeneration (Table 2).

**Table 2:** Functional enrichment of GO terms and KEGG pathways in chronic rat epilepsy gene expression

Term	Count	*p* value	Term	Count	*p* value
GO terms					
GO: molecular function			GO: biological process		
GO: 0003779—actin binding	27	1.8E−04	GO: 0006955—immune response	50	1.8E−07
GO: 0004714—transmembrane receptor tyrosine kinase activity	10	1.1E−03	GO: 0043067—regulation of programmed cell death	63	4.3E−05
GO: 0051015—actin filament binding	8	6.9E−03	GO: 0007610—behavior	44	5.2E−05
GO: 0016563—transcription activator activity	25	1.1E−02	GO: 0016477—cell migration	31	6.7E−05
GO: 0005272—sodium channel activity	6	1.3E−02	GO: 0007155—cell adhesion	46	9.6E−05
GO: 0022890—inorganic cation transmembrane transporter activity	15	1.4E−02	GO: 0030100—regulation of endocytosis	11	1.3E−03
GO: 0005216—ion channel activity	28	1.5E−02	GO: 0060627—regulation of vesicle-mediated transport	15	2.8E−03
GO: 0005261—cation channel activity	22	1.8E−02	GO: 0050804—regulation of synaptic transmission	19	3.6E−03
GO: 0005262—calcium channel activity	9	2.3E−02	GO: 0048167—regulation of synaptic plasticity	12	4.1E−03
GO: 0005244—voltage-gated ion channel activity	16	3.1E−02	GO: 0048666—neuron development	31	7.6E−03
KEGG pathways					
Downregulated genes			Upregulated genes		
rno04020: calcium signaling pathway	15	2.2E−05	rno04610: complement and coagulation cascades	14	1.4E−06
rno04010: MAPK signaling pathway	17	1.2E−04	rno00190: oxidative phosphorylation	16	1.6E−04
rno04360: axon guidance	9	4.9E−03	rno05012: Parkinson’s disease	15	7.8E−04
rno04730: long-term depression	6	1.2E−02	rno04612: antigen processing and presentation	11	1.5E−03
rno04720: long-term potentiation	6	1.3E−02	rno04650: natural killer cell mediated cytotoxicity	11	3.9E−03
rno04912: GnRH signaling pathway	7	1.4E−02	rno05016: Huntington’s disease	15	1.1E−02
rno04070: phosphatidylinositol signaling system	6	1.5E−02	rno04514: cell adhesion molecules (CAMs)	12	2.2E−02
rno04144: endocytosis	10	2.4E−02	rno05010: Alzheimer’s disease	14	3.2E−02

*GO* gene ontology, *KEGG* Kyoto encyclopedia of genes and genomes, *count* number of genes from our data set contributing to GO term and KEGG pathway enrichment, *rno* Rattus Norvegicus

Furthermore, we observed a large number of seizure-related genes [40] to be differentially expressed in PILO animals, including voltage-gated calcium, potassium or sodium channels (Cacng2, Itpr1, Kcna1, Kcna2, Kcnq2, Scn4b, Scn8a), calcium pumps (Atp2a2), neurotransmitter receptors (Gabrd), G-protein-coupled receptors (Gpr56) as well as components of the cytoskeleton (Gfap), proteins implicated in cell adhesion (Pcdh19), transcription factors (Tgif1) and metabolic enzymes (Ndufa2, Npc2) shown in Table 3. We also identified genes, which have not been previously associated with brain function, seizure generation or epilepsy-related processes.

**Table 3:** Differential gene expression of epilepsy-related genes

Gene ID	Gene name	Seizure-related disorder	Ensembl transcript ID	logFC	*p* value	FDR
Scn4b	Sodium channel, voltage-gated, type IV, beta	Long QT syndrome; Jervell-Lange Nielsen syndrome	ENSRNOT00000030152	−2.2	5.9E−13	5.3E−10
Gfap	Glial fibrillary acidic protein	Alexander disease	ENSRNOT00000034401	1.7	1.2E−09	4.9E−07
Npc2	Niemann-Pick disease, type C2	Niemann-Pick disease	ENSRNOT00000016076	1.2	9.6E−07	1.8E−04
Itpr1	Inositol 1,4,5-triphosphate receptor, type 1	Itpr1^−/−^ with seizure phenotype; Spinocerebellar ataxia	ENSRNOT00000009288	−1.2	1.1E−06	2.0E−04
Pcdh19	Protocadherin 19	Early infantile epileptic encephalopathy	ENSRNOT00000042335	−1.2	9.2E−06	1.2E−03
Gabrd	Gamma-aminobutyric acid (GABA) A receptor, delta	GEFS+; IGE; JME	ENSRNOT00000022246	−0.9	1.3E−04	8.9E−03
Kcna1	Potassium voltage-gated channel, shaker-related subfamily, member 1	Episodic ataxia; partial epilepsy	ENSRNOT00000026731	−1.1	1.9E−04	1.1E−02
Tgif1	TGFB-induced factor homeobox 1	Holoprosencephaly	ENSRNOT00000021534	1.5	2.7E−04	1.5E−02
Cacng2	Calcium channel, voltage-dependent, gamma subunit 2	Cacng2^−/−^ with seizure phenotype; Absence epilepsy; mental retardation	ENSRNOT00000008414	−0.9	1.5E−03	4.8E−02
Ndufa2	NADH dehydrogenase (ubiquinone) 1 alpha subcomplex, 2	Leigh syndrome	ENSRNOT00000023811	0.9	1.7E−03	5.0E−02
Scn8a	Sodium channel, voltage gated, type VIII, alpha subunit	Cerebellar atrophy, ataxia and mental retardation	ENSRNOT00000008160	−1.0	2.1E−03	5.8E−02
Gpr56	G protein-coupled receptor 56	Polymicrogyria	ENSRNOT00000020921	−0.8	2.4E−03	6.1E−02
Kcnq2	Potassium voltage-gated channel, KQT-like subfamily, member 2	BFNS; Early infantile episodic encephalopathy	ENSRNOT00000016574	−1.0	2.5E−03	6.4E−02
Atp2a2	ATPase, Ca++ transporting, cardiac muscle, slow twitch 2	Darier-White disease	ENSRNOT00000067047	−0.9	3.4E−03	7.4E−02
Kcna2	Potassium voltage-gated channel, shaker-related subfamily, member 2	Kcna2^−/−^ with seizure phenotype; Episodic ataxia	ENSRNOT00000050149	−0.9	3.9E−03	7.9E−02

According to Gene Cards, JaxMice Database and Lemke et al. [40]

*logFC* log2 fold change, leading sign indicates direction of change (+, increase; −, decrease), *FDR* false discovery rate, *BFNS* benign familial neonatal seizures, *GEFS+* generalized epilepsy with febrile seizures plus, *IGE* idiopathic generalized epilepsy, *JME* juvenile myoclonic epilepsy

Recent experimental results in human TLE detected altered expression of DNMT isoforms [79]. These enzymes are involved in the establishment or maintenance of genomic DNA methylation patterns [19, 55, 79]. Analysis of our mRNA-Seq data revealed no significant change in gene expression for the DNA methyltransferases Dnmt1, Dnmt3a and Dnmt3b in the PILO cohort. But chromatin structure and function may not exclusively be regulated through DNA methylation. We report differential gene expression of other key epigenetic enzymes and downstream effector proteins implicated in histone acetylation and methylation, as well as micro RNA processing and ATP-dependent chromatin remodeling (manual inspection and GO term analysis, cut-off *p* < 0.01, Table 4). These results suggest that different epigenetic pathways could be involved in the pathogenesis of epilepsy and maintenance of the chronic disease state.

**Table 4:** Epigenetic signature in gene expression

GeneID	Gene name	Function	Ensembl transcript ID	logFC	*p* value	FDR
Gadd45a	Growth arrest and DNA-damage-inducible 45 alpha	DNA *de*methylation, base excision repair	ENSRNOT00000007698	1.42	2.2E−06	3.6E−04
Apobec1	Apolipoprotein B mRNA editing enzyme, catalytic polypeptide 1	DNA *de*methylation, cytidine deaminase, base excision repair	ENSRNOT00000020735	1.27	3.5E−04	1.7E−02
Eif2c1	Eukaryotic translation initiation factor 2C, 1; argonaute 1	miR pathway, inhibition of translation	ENSRNOT00000037728	−0.94	8.6E−04	3.4E−02
Ncoa1	Nuclear receptor coactivator 1	HAT activity towards H3 and H4, participates in chromatin remodeling and recruitment of general transcription factors	ENSRNOT00000005782	−0.89	1.0E−03	3.7E−02
Ezh1	Enhancer of zeste homolog 1	HMT activity, H3K27 specific	ENSRNOT00000027640	−0.87	1.1E−03	3.8E−02
Zmynd8	Zinc finger, MYND-type containing 8	Chromatin remodeling factor	ENSRNOT00000025932	−0.80	1.7E−03	5.0E−02
Mthfs	5, 10-methenyltetrahydrofolate synthetase	Folate metabolism and transmethylation pathway	ENSRNOT00000039850	1.14	3.9E−03	7.9E−02
Mll1	Myeloid/lymphoid or mixed-lineage leukemia 1	HMT activity, H3K4 specific	ENSRNOT00000020573	−0.87	5.3E−03	9.4E−02
Nrip1	Nuclear receptor interacting protein 1	Serves as a scaffold for both DNMT and HMT activities to inhibit gene transcription	ENSRNOT00000002152	−0.82	5.4E−03	9.5E−02
Cbx5	Chromobox 5	HP1 homolog, binding of 5mC	ENSRNOT00000055289	0.72	5.5E−03	9.6E−02

According to GO terms and manual inspection

*logFC* log2 fold change, leading sign indicates direction of change (+, increase; −, decrease), *FDR* false discovery rate, *DNMT* DNA methyltransferase, *HAT* Histone acetyltransferase, *HMT* Histone methyltransferase, *miR* microRNA, *5mC* 5-methyl-cytosin

### DNA methylation is inversely correlated with gene expression in chronic epileptic rats

**Fig. 4:**
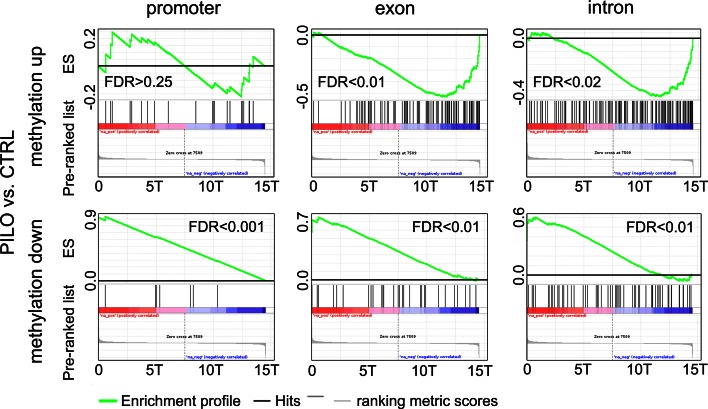
Gene set enrichment analysis (GSEA) of methylated promoters, TSS and gene bodies were performed against the rank of our mRNA-Seq data from same samples. GSEA was separately performed for gene sets showing increased or decreased methylation in chronic rat epilepsy. A strong correlation (FDR < 0.25 was considered significant) was observed between gene body methylation and gene repression, and conversely, between loss of intragenic methylation and activated gene expression. Promoter hypermethylation was not associated with gene repression. *FDR* false discovery rate, *T* thousand, *CTRL* sham injected, healthy controls, *PILO* pilocarpine injected, chronic epileptic animals. Supplement Table 1 contains comprehensive GSEA statistics

Next we determined whether DNA methylation changes targeted gene expression in our model of chronic epilepsy. A Gene Set Enrichment Analysis (GSEA) was performed [72] using the ranked mRNA results and sets of differentially methylated genes as described above. In PILO animals, differential DNA methylation targeted 1,180 specific gene loci (overlap ≥1 bp with annotated promoter, TSS or gene body). Generally, these DNA methylation changes were not associated with alterations in gene expression (*n* = 930, 79 %). However, for 250 gene loci (21 %) a strong correlation between differential DNA methylation and gene expression was identified. DNA hypermethylation of gene bodies, introns as well as exons was clearly associated with gene silencing in chronic rat epilepsy, but we did not find hypermethylation of gene promoters correlated with gene suppression (Fig. 4 upper panel, and Supplement Table 1). In contrast, decreased methylation was associated with increased gene expression and a feature of exons, introns, TSS and gene promoters (Fig. 4 lower panel, and Supplement Table 1).

To understand biological pathways targeted by altered gene expression patterns and DNA methylation, we performed Functional Annotation Clustering using DAVID. This analysis identified enrichment of genes implicated in cytoskeleton organization, immune response and inflammation, neuronal development and differentiation, cell adhesion, as well as cell projection. Furthermore, we observed changes in genes involved in calcium signaling, DNA binding and transcription, programmed cell death, and synaptic transmission (enrichment score for all clusters >1.5). Taken together, GSEA would support a biological relevance of DNA methylation changes in our experimental epilepsy model.

### Validation of candidate genes in epilepsy

**Fig. 5:**
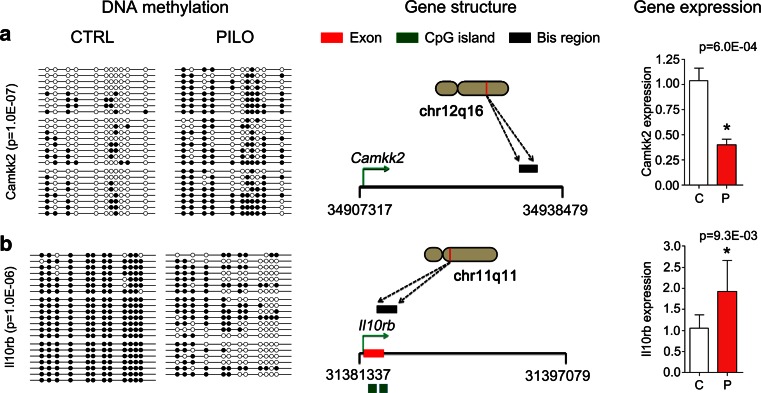
*Left panel* showing bisulfite sequencing results (Bis-Seq, *n* = 3). *White dots* represent unmethylated and *black dots* methylated CpGs. *Middle panel* summarizing schematic gene structure with TSS (*green arrow*), chromosomal region and region covered in Bis-Seq. *Right panel* presenting gene expression data from RT-PCR (*n* = 5). *C*, *CTRL* control (*white bar*); *P*, *PILO* chronic epileptic animals (*red bar*). *Asterisks* indicate significance (unpaired two-tailed *t* test, *p* < 0.05). **a** Camkk2 showed hypermethylation and concomitant gene repression in PILO versus CTRL. **b** Hypomethylation of the Il10rb locus and increased gene expression could be confirmed in PILO versus CTRL

Next we sought to validate DNA methylation targeted gene expression. Bisulfite sequencing was used to verify differential methylation from Methyl-Seq in chronic rat epilepsy compared to healthy controls. To quantify differences in CpG methylation between PILO and CTRL, Mann–Whitney *U* test was performed. Fisher’s Exact Test was further calculated to analyze the independence of CpG methylation between two treatment groups at a particular CpG site. The schematic shown in Fig. 5 (middle panel) illustrates the gene and its chromosomal localization as well as the location of the amplicon examined using bisulfite sequencing. We confirmed the hypermethylation status of Camkk2 (Ca^2+^/Calmodulin-dependent protein kinase kinase 2, beta; RN4 genome assembly, chr12:34936408-34936667; Mann–Whitney *U* test *p* = 1E − 07; Fig. 5a, left panel; Supplement Table 2a), a key enzyme in Ca^2+^ signaling involved in hippocampus-dependent long-term memory, and hypomethylation of the Il10rb locus (interleukin 10 receptor, beta; RN4, chr11:31381431-31381722; Mann–Whitney *U* test *p* = 1E-06; Fig. 5b, left panel; Supplement Table 2b), an endogenous cytokine receptor involved in anti-inflammation and neuroprotection, in chronic rat epilepsy compared to healthy controls.

We further validated gene expression changes identified by mRNA-Seq using qRT-PCR. Camkk2 gene expression was significantly reduced in PILO animals (unpaired two-tailed *t* test, *p* = 6.0E-04; Fig. 5a, right panel) and consistent with increased gene methylation. Increased expression of Il10rb (unpaired two-tailed *t* test, *p* = 9.3E-03; Fig. 5b, right panel) was inversely correlated with a hypomethylation phenotype in chronic rat epilepsy. These results confirm gene expression changes mediated by DNA methylation, as identified in our genome-wide sequencing approaches, using independent assays such as bisulfite sequencing and qRT-PCR, respectively.

### Ketogenic diet ameliorates seizure-induced DNA methylation in chronic rat epilepsy

Next we explored whether anti-epileptic treatment could change DNA methylation mediated gene expression in our experimental animal model, because medically refractory epilepsies frequently respond to strict dietary regimens. Indeed, the ketogenic diet (KD), a high-fat, moderate protein diet with low carbohydrate content has been implicated in regulating gene expression by modifying chromatin structure [68]. To test our hypothesis that a ketogenic diet could alter DNA methylation mediated gene expression we fed a subset of animals with a non-calorie restricted KD (PILO + KD) immediately following SE. KD treatment had no effect on SE as the initial precipitating injury. We tested ketosis as reliable parameter that the KD changed the animals’ metabolism 2 weeks after initial treatment using standard reagent strips for urine analysis, i.e., “ketosticks” (Bayer, Leverkusen, Germany; Supplement Fig. 2). To assess any effect of dietary treatment on clinical phenotype, behavioral seizures were continuously monitored during the entire study period of 12 weeks after SE. Compared to SE-experienced animals fed a standard diet, KD administration did not result in differences regarding latency period or mean severity and duration of clinical seizures. A significant difference, however, was detected according to seizure frequency per week (paired two-tailed *t* test, *p* = 4.0E-04; mean ± SEM: PILO = 24.5 ± 4.7, PILO + KD = 6.9 ± 1.9) with a reduction in seizure burden upon KD treatment and apparent delay in the chronification of the disease (Supplement Fig. 1b). Recordings from subdural electrodes and limited sample numbers with vEEG-monitoring did not allow meaningful quantification of KD effects on EEG data, but comparison of Racine stage 5 clinical seizures and baseline EEG provided no evidence for treatment specific differences between PILO and PILO + KD (Supplement Fig. 1c).

**Fig. 6:**
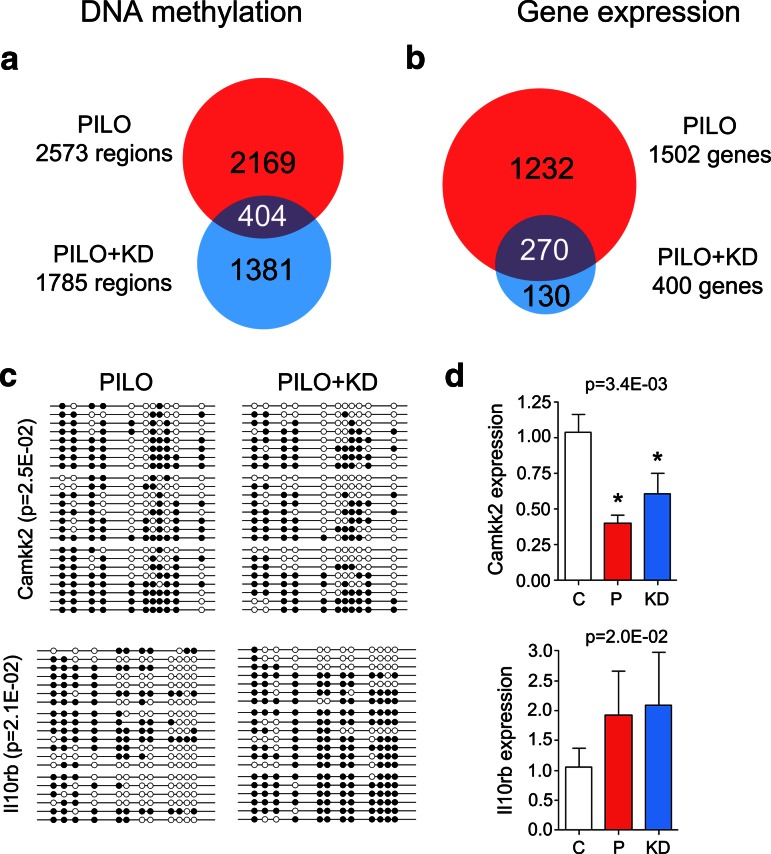
Comparison of differential DNA methylation and gene expression patterns in pilocarpine injected, epileptic animals receiving anti-convulsive ketogenic diet (PILO + KD, *blue*) or no treatment (PILO, *red*). The KD treatment partially ameliorated molecular changes associated with chronic rat epilepsy. Effects were more pronounced on a genomic scale than at certain loci of selected candidate genes. **a** Venn diagram displaying overlap in differential DNA methylation between PILO and PILO + KD compared to CTRL. KD-treated animals showed a distinct genomic methylation profile compared to untreated chronic rat epilepsy. Administration of the KD seemed to have rescued a majority of affected loci. **b** Venn diagram displaying overlap in differential gene expression between PILO and PILO + KD compared to CTRL. KD-treated animals showed a distinct gene expression pattern compared to untreated chronic rat epilepsy. A majority of differentially expressed genes in PILO were rescued upon KD treatment. Genes exclusively expressed in PILO + KD may have contributed to adverse side effects. **c** Bisulfite sequencing results. Camkk2 hypermethylation in PILO animals was significantly reduced by KD treatment (Mann–Whitney *U* test, *p* < 0.05). Further, hypomethylation of the Il10rb locus in PILO was reversed in KD + PILO. White dots represent unmethylated and black dots methylated CpGs. **d** Gene expression of Camkk2 and Il10rb in PILO and PILO + KD. KD treatment partially rescued Camkk2 gene expression, but had no significant effect on Il10rb. *C*, *CTRL* control (*white bar*); *P*, *PILO* chronic epileptic animals (*red bar*); *KD*, *PILO* *+* *KD* pilocarpine injected animals receiving anti-convulsive ketogenic dietary treatment (*blue bar*). *Asterisks* indicate significance (univariate one-way ANOVA followed by Bonferroni post hoc test, *p* < 0.05)

We analyzed differential methylation in epileptic animals assigned to anti-epileptic dietary treatment. Pairwise analysis of PILO + KD versus CTRL identified 1,785 loci that were differentially methylated. Thereof, 1,003 loci associated with hypermethylation and 782 regions with hypomethylation. Interestingly, the targeted genomic regions showed limited overlap with regions previously identified in our untreated epileptic PILO animals (Fig. 6a, blue). In KD-treated animals we observed strong reduction in DNA methylation at gene bodies as well as intronic and exonic regions. These results clearly show that genomic DNA methylation patterns of CTRL and PILO animals are distinguishable from the PILO + KD group. All animals from the PILO and PILO + KD group experienced convulsive seizures within 48 h preceding their termination (unpaired two-tailed *t* test, *p* > 0.05), suggesting that differences in methylation between groups were not dependent on the time point of their last seizure.

Gene expression profiling in KD-treated rats identified 400 differentially regulated genes compared to over 1,500 aberrantly expressed genes in PILO (cut-off *p* < 0.01; Fig. 6b). Gene expression again was inversely correlated with genomic DNA methylation patterns (Supplement Table 1). To determine whether expression changes were associated with the anti-seizure phenotype and not induced by the diet, we compared our mRNA-Seq data with a recently described gene expression profile (GEO Data Set 954) derived from healthy, non-epileptic rats receiving a KD [10]. Consistent with our hypothesis that an anti-convulsive dietary treatment could alter DNA methylation mediated gene expression in rat epilepsy there was no correlation between the reference CTRL + KD and our PILO + KD (data not shown).

Validation experiments of our candidate genes, Camkk2 and Il10rb, provide additional evidence that the ketogenic diet may be useful to ameliorate aberrant seizure-associated DNA methylation (Mann–Whitney *U* test, *p*_Camkk2_ = 2.5E-02 and *p*_Il10rb_ = 2.1E-02; Fig. 6c) and concomitant gene expression (one-way ANOVA with Bonferroni post hoc test, *p*_Camkk2_ = 3.4E-03 and *p*_Il10rb_ = 2.0E-02; Fig. 6d).

### Gene expression profiles reveal further regulatory pathways implicated in chronic epilepsy

Gene expression is coordinated by complex regulatory mechanisms that involve transcription factor binding and chromatin modification including DNA methylation. To explore mechanisms that serve to regulate coordinated gene expression changes in chronic rat epilepsy we examined our mRNA-Seq data for changes consistent with transcription factor signaling using GSEA. We found that genes downregulated in our epilepsy model (PILO and PILO + KD) shared binding motifs for neuron restrictive silencing factor (Nrsf) and suppressor of zeste 12 (Suz12). The former is a major transcriptional regulator of neuronal gene expression that recruits Dnmts and Hdacs, and the latter is a central component of the Polycomb repressor complex 2 (Prc2). Downregulated genes from PILO and PILO + KD animals with Suz12/Prc2 binding capacity were generally hypomethylated, consistent with a recent study [24]. Examination of upregulated genes in PILO and PILO + KD animals identified that nuclear factor kappa B (Nfkb), K(lysine) acetyltransferase 2 (Kat2a), zinc finger ZZ domain containing 3 (Zzz3), general transcription factor IIB (Gtf2b) and p300 binding motifs were commonly shared. Nfkb is a rapidly acting primary transcription factor that plays a key role in regulating inflammation and immune response also in epilepsy [74]. Kat2a, Zzz3 and p300 are distinguished transcriptional activators, which share histone acetyltransferase activity [70]. Taken together, our results indicate that important transcription factors, which serve to function in chromatin modification, together with DNA methylation may participate in regulating common patterns of gene expression in chronic epilepsy.

## Discussion

This is the first report describing genome-wide changes in DNA methylation in the chronic stage of rat TLE. Dissecting rat hippocampal tissue, mRNA sequencing identified deregulation of seizure- and epilepsy-related genes, metabolic and key epigenetic enzymes or regulators. DNA methylation was found to be inversely correlated with gene expression, and candidate genes were validated using bisulfite sequencing and real-time PCR. Ketogenic diet, a well recognized anti-epileptic treatment in children with severe, chronic epilepsy [13, 54], attenuated seizure burden, delayed chronification of the disease and partially rescued the DNA methylation and corresponding gene expression phenotype.

DNA methylation not only regulates cell fate determination and maturation in the brain but also plays a role for the induction of activity-dependent synaptic plasticity, memory and cognition [20, 41, 42, 60]. The importance of epigenetic tuning of higher order brain function is emphasized by a growing number of neurological diseases that associate with alterations in DNA methylation, including autism spectrum disorders, schizophrenia, Alzheimer’s disease, brain tumors, spinal muscular atrophy and, more recently, epilepsy [35]. So far, epigenetic gene regulation in epileptogenesis had only been investigated for individual candidate genes (e.g., Reelin, BDNF, GluR2) and/or focused on the very early stage of the disease, during or immediately following SE [36, 47, 53, 73]. Our present study is unique for its simultaneous DNA methylation and gene expression profiling, suggesting DNA methylation as potential molecular pathomechanism in chronic epilepsy contributing to the deregulation of multiple genes.

We observed specific DNA methylation signatures, which readily discriminate chronic epileptic PILO from reference CTRL and PILO + KD animals using hierarchical cluster analysis. Genome-wide unsupervised clustering of an epigenetic mark could distinguish epileptic from non-epileptic animals. We observed more frequent hypermethylation of genes in rats with chronic epilepsy. Indeed, changes in DNA methylation were predominantly located at CGIs within gene bodies, and generally did not target gene promoters. A similar pattern of gene body methylation was recently described in the healthy rat and human methylome [15, 67]. In this study, DNA methylation was inversely correlated with gene expression. Targeted validation experiments supported this finding, providing some evidence for the biological significance of seizure-associated DNA methylation changes. Both hyper- and hypomethylation events were detected with subsequent gene repression or activation. The calcium/calmodulin-dependent protein kinase kinase 2, beta (Camkk2) was down-regulated in our chronic epileptic animals when compared to controls and showed intragenic hypermethylation. Camkk2 is a key enzyme in calcium signaling, which mainly phosphorylates Camk1 and Camk4 as well as AMP-activated protein kinase (Ampk). It further seems to be involved in hippocampal activation of the transcription factor cAMP response element binding protein (Creb1) and downstream immediate early genes [62]. Camkk2 is highly expressed in the brain, and involved in long-term potentiation and hippocampus-dependent memory formation [56], granule cell development [37] and cortical axon elongation [2]. Thus, altered expression of Camkk2 may contribute to seizure-associated memory impairment. In contrast, the Il10rb was hypomethylation in the chronic epileptic PILO cohort and this was consistent with increased gene expression. Il10rb belongs to the cytokine receptor family and is an essential accessory chain for the active IL-10 receptor complex. The localization of the IL-10 receptor in five major regions of the rat brain including hippocampus supports a central role in inflammation signaling [76]. Interestingly, the expression of IL-10 is elevated during the course of most major diseases in the CNS including TLE and promotes survival of neurons and all glial cells in the brain by blocking the effects of proapoptotic cytokines and promoting expression of cell survival signals [32, 71]. Therefore, overexpression of IL10rb in rat epilepsy could be a compensatory mechanism to limit brain damage following seizures.

Genome-wide profiling also clearly identified methylation changes that did not correspond with gene expression changes. Transcriptional regulation is dependent on several mechanisms that include chromatin remodeling, microRNAs, histone modifications as well as transcription factor binding together with DNA methylation. Consistent with this, we identified differential gene expression for a number of epigenetic enzymes (Table 4) as well as binding motifs for key transcriptional activators (Nfkb, Kat2a, Zzz3, Gtf2b, p300) and repressors (Nrsf, Suz12) overrepresented in our gene sets. Interestingly, some transcription factors also function to regulate chromatin structure [3, 29, 46, 70]. Our analysis highlighted Prc2 interaction conferred by Suz12. Previous studies suggest that the Prc2 complex may serve as a recruiting platform also for Dnmts, thereby linking two epigenetic repression systems [66, 75]. Our data do not support this interplay as downregulated genes with Suz12 response element did not show increased DNA methylation. This finding is in line with recent studies, where authors showed that trimethylation of histone 3 lysine 27 (H3K27) and DNA methylation are mutually exclusive particularly at CGIs [11, 24]. Taken together, our in silico analysis of common transcription factor binding motifs supports a model of transcriptional regulation in chronic rat epilepsy, where gene activation is conferred by histone acetylation and gene suppression mediated by antagonistic effects of DNA and histone methylation.

In this study, DNA methylation provided a strong signal to separate chronic epileptic rats from healthy controls, but it remained unsolved, whether the presence of spontaneous seizures (epilepsy) led to altered methylation, or if altered methylation influenced seizures. To test the first hypothesis, we analyzed whether early administration of a high-fat, high-protein, low-carbohydrate ketogenic diet (KD) would partially rescue the seizure-associated and gene regulating DNA methylation changes in our rat TLE model. The KD is a clinically effective treatment in children with epilepsy and severe cognitive impairment, pharmacoresistant to conventional and even newer anti-convulsant medications [54]. We show that KD administration in our epilepsy model partially attenuated seizure burden, delayed disease progression and interfered with aberrant seizure-related genomic and locus specific alterations in DNA methylation and gene expression. How the ketogenic diet works to control or attenuate seizures remains poorly understood [13, 48]. The mechanisms proposed include metabolic changes (restricting glycolysis, increasing fatty acid oxidation, mitochondrial respiration and ATP synthesis), increased GABAergic inhibition, modulation of oxidative stress and neuroprotection [63]. It is most likely that described mechanisms work complementary, and we suggest that direct or indirect induction of epigenetic changes may add to this complexity [23, 49, 68].

Consistent with our hypothesis that gene expression changes associated with the anti-seizure phenotype, we compared a differential gene list derived from a recent study involving also non-epileptic KD-treated control animals [10] with the rank of our PILO + KD cohort. Comparison of data sets showed no correlation of gene expression patterns with diet, supporting the idea that the anti-convulsive properties of the KD contribute to DNA methylation mediated gene expression changes in PILO + KD. Future studies could determine whether DNA methylation affects seizures using known DNMT inhibitors. Since they have not been tested thoroughly this remains speculative. The proposed relationship between neuronal hypersynchronous activity and gene regulation mediated by epigenetic changes could also be explored beyond the present study design to address timing and signal transition from the acute to the chronic disease phase as well as cell-specificity of epigenetic events. We cannot exclude that regional differences in DNA methylation and gene expression in our study may have been masked in part using whole hippocampus. Prospective utilization of microdissected homogenous cell populations from specific hippocampal subfields will help to decipher where exactly seizure-related DNA methylation changes occur (e.g., glia or the neuronal subpopulations of pyramidal and granular cells), and which of the detected signals in the present study were only related to cell composition effects in the different samples.

In conclusion, the characterization of mechanisms underlying epigenetic changes in the chronic epileptic brain should lead to a better understanding of disease. We consider that our genome-wide analyses provide a comprehensive profile of DNA methylation mediated gene expression changes, and may help to identify new regulatory targets in epilepsy, which again could be addressed by novel treatment strategies. By defining the principal events mediating epigenomic changes it is anticipated that novel approaches will be developed to inhibit, attenuate or reverse the persistent deleterious consequences of seizures in the epileptic brain.

## Electronic supplementary material

Below is the link to the electronic supplementary material.

**Supplement Figure 1a:** Graphical summary of body weight development. Epileptic animals show slightly reduced body weight compared to CTRL (black) No difference in development is apparent between PILO (red) and PILO+KD (blue; two-way ANOVA, p>0.05). **1b:** KD treatment significantly reduced seizure burden and delayed disease progression according to seizure frequency per week (paired two-tailed t-test, *p*<0.05). CTRL – sham injected, healthy controls; PILO – pilocarpine injected, chronic epileptic animals; PILO+KD – pilocarpine injected animals receiving anti-convulsive ketogenic dietary treatment. **1c:** EEG data shows no difference in ictal or interictal abnormalities between PILO and PILO+KD animals. R5 – clinical seizure, class 5 according to Racine scale.

**Supplement Figure 2:** Neuronal cell counts were performed in video-monitored reference animals (CTRL, PILO and PILO+KD; n=3) terminated 4 weeks following initial treatments using a previously established protocol for the classification of hippocampal cell loss in human TLE [8, 9] adapted to our rat TLE model. Cell counts were performed in a defined localization of hippocampal tissue from the medial level (Bregma -3.30 to -4.16 mm). Immunohistochemically stained neuronal cell bodies (NeuN) were tagged on the computer screen and manually counted in 10 consecutive ultra thin sections (3 µm) within rat hippocampal sectors CA1, CA3a and CA3c in 4 adjacent visual fields at 20× objective magnification, representing 62,500 μm², along the pyramidal cell layer. Granule cells of the dentate gyrus were counted at 40× objective magnification in 10 adjacent visual fields, representing 100,000 µm^2^, along the lower (infragranular) limb of the dentate gyrus. Hilar cells were counted at 40× objective magnification in 3 adjacent visual fields, representing 30,000 µm^2^. For all neuronal cell counts the mean number of neurons/ mm^2^ was calculated. The protocol for TIMM staining was previously described [5]. In short, following preparation the hemisphere was instantly put in ice cold PBS and cut into 400 µm coronal sections (Vibratome 1000S, Leica microsystems, Wetzlar, Germany). For Timm’s stain, coronal hippocampal 400 μm slices were sequentially incubated in 0.1 % sodium sulfide for 10 min, 0.3 % glutaraldehyde in 0.15 M phosphate buffer for 10 min, 70 % ethanol for at least 1 d, and 30 % sucrose in 0.15 M phosphate buffer overnight at 4 °C. The 400 μm slices were then cut at 20 μm on a cryostat and air-dried for 1 h. After rinsing in 0.15 M phosphate buffer, mounted sections were incubated in developing solution for 30 min until the molecular/ supragranular layer in the dentate gyrus (DG) was clearly stained. Reaction was stopped using sodium thiosulfate solution and sections were counterstained with toluidine blue. Our animal model revealed altered neuronal cell densities in hippocampal subfields CA1 (univariate one-way ANOVA and Bonferroni post hoc test, mean number of cells/mm^2^ ± SEM: CTRL = 944 ± 22; PILO = 643 ± 24; PILO + KD = 775 ± 21; *p* = 1.0E-04), CA3a (CTRL = 780 ± 17; PILO = 499 ± 26; PILO + KD = 522 ± 12; *p* = 1.4E-02), hilus (CTRL = 534 ± 16; PILO = 397 ± 20; PILO + KD = 369 ± 17; *p*=1.0E-04), but not in CA3c (CTRL = 674 ± 23; PILO = 665 ± 17; PILO+KD = 711 ± 13; *p* = 1.0E-01) or DG (CTRL = 4,862 ± 52; PILO = 5,108 ± 105; PILO + KD = 4,900 ± 117; *p* = 1.5E-01) of chronic epileptic rats with and without KD compared to controls (right panel). We did not detect complete neuronal loss, neither in CA3a nor CA1 (left panel). KD treatment partially improved pathological changes in our pilocarpine model, but did not completely diminish neurodegeneration. Timm staining revealed mossy fiber sprouting in the supragranular and inner molecular layer of the DG of chronic epileptic rats, but no differences between PILO and PILO + KD were apparent by visual inspection (middle panel).

**Supplement Table 1:** GSEA – gene set enrichment analysis; NES - normalized enrichment score; NOM p-val - nominal p-value; FDR - false discovery rate (FDR < 0.25 was considered significant).

**Supplement Table 2:** Camkk2 – Ca^2+^/Calmodulin-dependent protein kinase kinase 2, beta; Il10rb – Interleukin receptor 10, beta; CTRL – sham injected, healthy controls; PILO - chronic epileptic animals; RN4 – rat genome assembly; *p* < 0.05 was considered significant.

Supplementary material 1 (TIFF 990 kb)

Supplementary material 2 (TIFF 49377 kb)

Supplementary material 3 (DOC 36 kb)
