# Supplementary figures and images for: Deep sequencing reveals increased DNA methylation in chronic rat epilepsy
Source: Acta Neuropathol. 2013 Sep 5;126(5):741–56. doi: 10.1007/s00401-013-1168-8 (PMC3825532; doi:10.1007/s00401-013-1168-8)

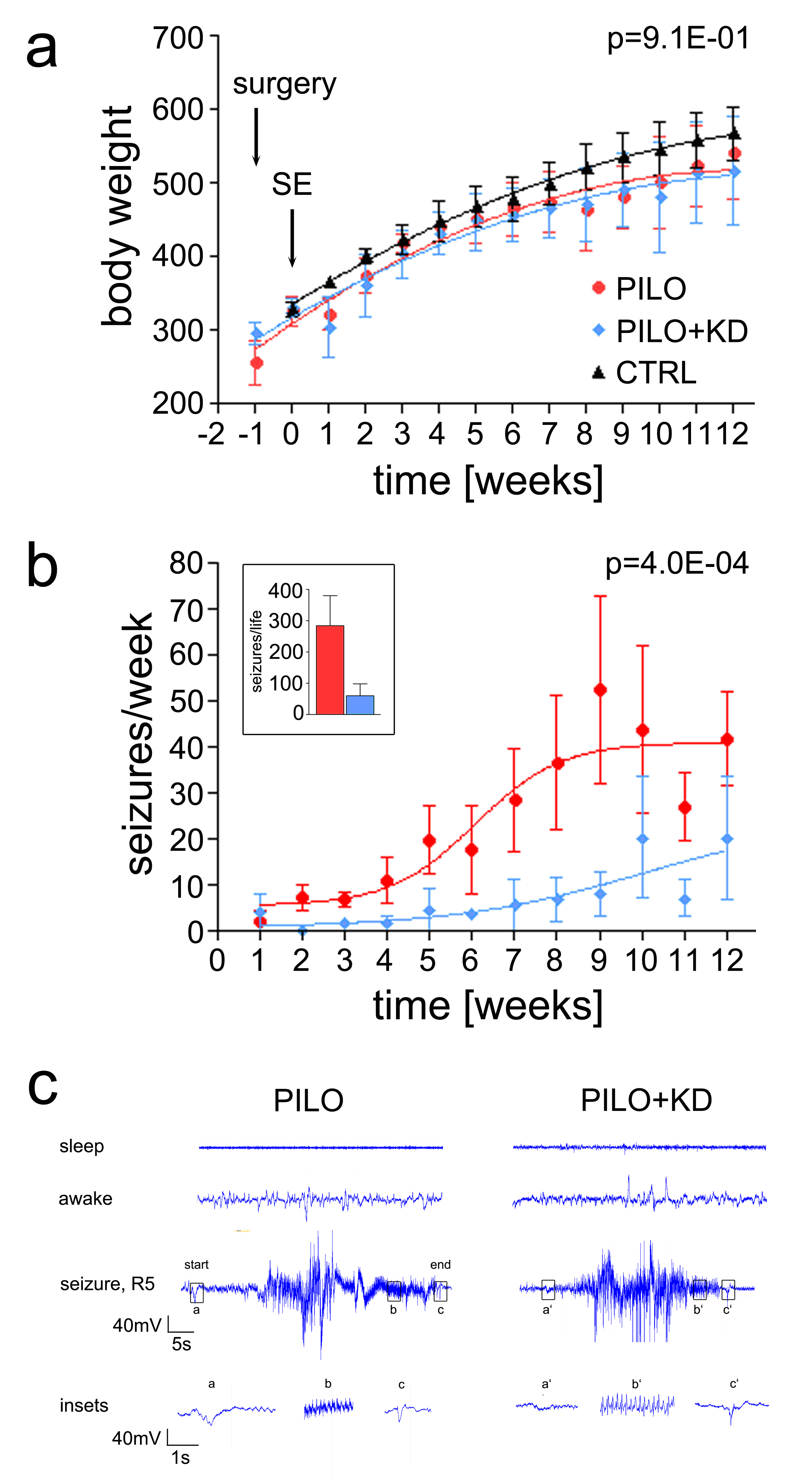

Supplement: Supplementary file 1 — Supplementary material 1 (TIFF 990 kb) [file 401_2013_1168_MOESM1_ESM.tif]

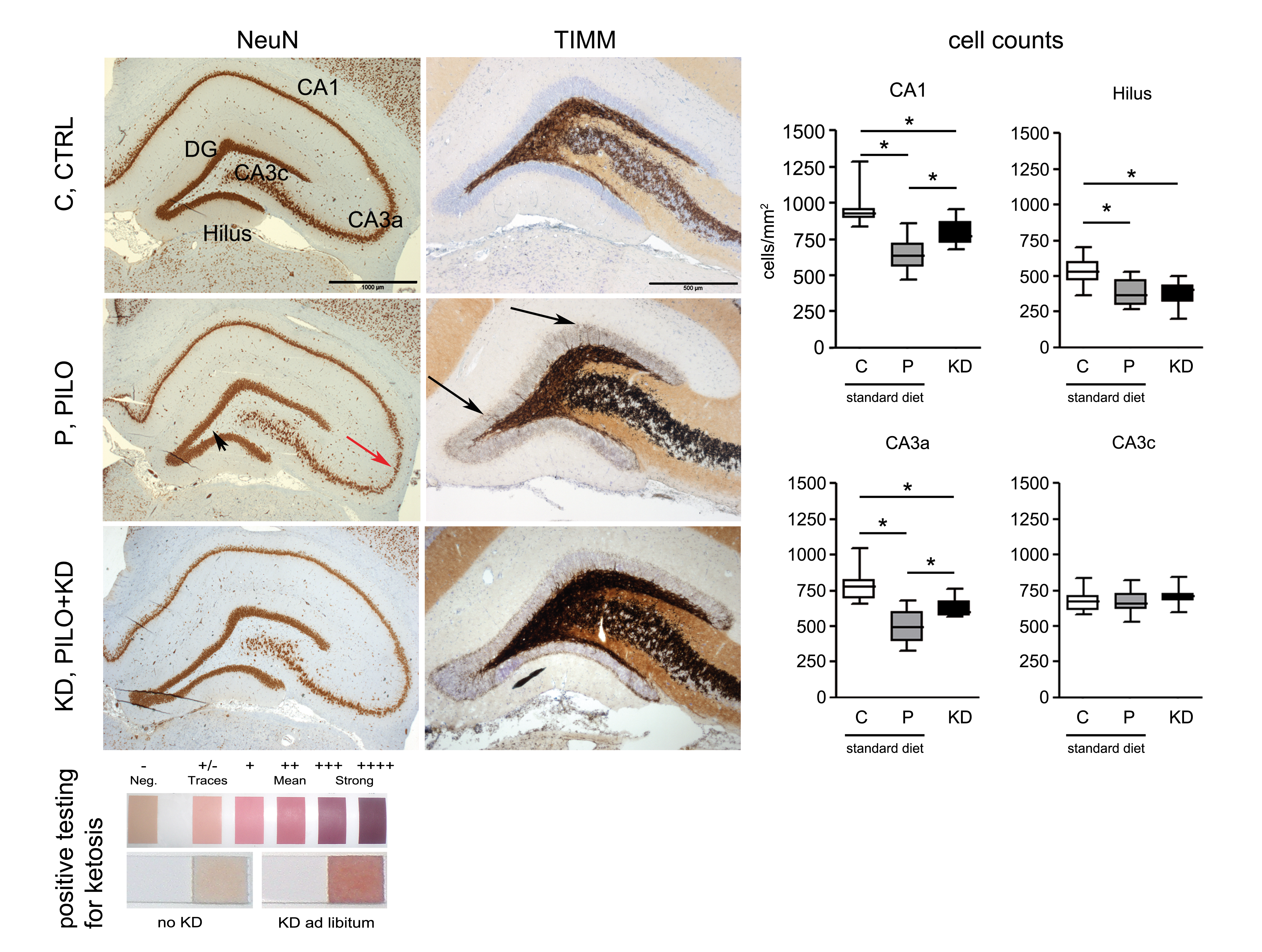

Supplement: Supplementary file 2 — Supplementary material 2 (TIFF 49377 kb) [file 401_2013_1168_MOESM2_ESM.tif]
