# Supplementary material for: Deep sequencing reveals increased DNA methylation in chronic rat epilepsy
Source: Acta Neuropathol. 2013 Sep 5;126(5):741–56. doi: 10.1007/s00401-013-1168-8 (PMC3825532; doi:10.1007/s00401-013-1168-8)
Supplement: Supplementary file 3 — Supplementary material 3 (DOC 36 kb) [file 401_2013_1168_MOESM3_ESM.doc]

**Supplement Table 1: Summary of GSEA statistics of methylated genomic features compared to mRNA-Seq results**

**Supplement Table 2a: Statistical analysis of Camkk2 methylation**

**Supplement Table 2b: Statistical analysis of Il10rb methylation**
